# Temporal Multi-Omics Reveals Microbial and Metabolic Succession Following Astilbin Treatment in a Human Colonic Model

**DOI:** 10.3390/nu18162616

**Published:** 2026-08-10

**Authors:** Tingwei Wang, Chang Liu, Jian Ji, Shuang Zhang, Shengfang Wu, Bangen Xia, Ruowei Xia, Nian Qu, Maiqiu Wang, Lei Zhang, Yongli Ye

**Affiliations:** 1 College of Biological & Chemical Engineering, Zhejiang University of Science and Technology, Hangzhou 310023, China; wangtingwei@zust.edu.cn (T.W.);; 2 Zhejiang Key Laboratory of Biomedical Intelligent Computing Technology, Hangzhou 310023, China; 3 School of Food Science and Technology, International Joint Laboratory on Food Safety, Synergetic Innovation Center of Food Safety and Quality Control, Jiangnan University, Wuxi 214122, China; 4 Instrumental Analysis and Laboratory Animal Center, Jiangnan University, Wuxi 214122, China; 5 Ningbo Xiabang New Pharmaceutical Technology Co., Ltd., Ningbo 315000, China

**Keywords:** astilbin, gut microbiota, time-series multi-omics, microbial ecological succession, 3-indolepropionic acid, inflammation resolution

## Abstract

**Background:** Astilbin is a bioactive flavonoid with documented anti-inflammatory properties; however, its sustained interactions with the gut microbiota remain poorly understood. Fecal samples from three healthy donors were pooled and fermented with astilbin in an in vitro human colonic model over 7 days. This exploratory study aimed to characterize the temporal ecological shifts associated with prolonged astilbin exposure. **Methods:** Time-resolved 16S rRNA gene sequencing, PICRUSt2 functional prediction, BugBase phenotypic inference, and pseudo-targeted metabolomics were integrated to track microbial-metabolic dynamics. **Results:** Astilbin exposure was associated with a highly coordinated, three-stage microbial succession. Day 3 (D3) emerged as a putative inflection point, where the enrichment of pioneer degraders (*Flavonifractor*, *Bacteroides*) was temporally correlated with the appearance of polyphenol cleavage intermediates. This transition featured an early decrease in markers of proteolytic fermentation alongside a transient in vitro lipid-stress response. By D7, the community shifted toward a stable configuration enriched in butyrogenic taxa (*Roseburia*, *Subdoligranulum*, *Megamonas*), with progressive depletion of potentially opportunistic pathogens (*Escherichia-Shigella*). Multi-omics integration suggested that these structural successions were strongly associated with marked metabolic shifts. Inflammatory lipid markers (e.g., leukotriene B4) showed a characteristic “D3-burst/D7-clearance” pattern, whereas potentially barrier-protective metabolites, particularly 3-indolepropionic acid (3-IPA, log_2_FC = 2.00) and urolithin B (log_2_FC = 1.18), accumulated substantially. **Conclusions:** This exploratory study provides valuable high-resolution insights into astilbin’s potential as a dynamic ecological modulator. It outlines a temporal framework illustrating how the gut microbiota may shift from proteolytic fermentation toward 3-IPA-associated homeostasis. Although limited by a pooled fecal model and the absence of a vehicle control, these hypothesis-generating findings offer a solid foundation for future in vivo studies and mechanistic validations across diverse human cohorts.

## 1. Introduction

The gut microbiota functions as a highly dynamic bioreactor, encoding an extensive repertoire of metabolic enzymes that transform dietary components into diverse bioactive small molecules [1]. Microbially derived metabolites, including short-chain fatty acids (SCFAs), secondary bile acids, and tryptophan derivatives such as indoles, act as key signaling mediators that support intestinal barrier integrity and regulate host immune homeostasis [2,3,4]. By contrast, microbial dysbiosis can promote unfavorable metabolic shifts, including enhanced proteolytic fermentation and excessive production of pro-inflammatory lipid mediators and toxic bile acids. These alterations contribute to chronic intestinal inflammation and systemic metabolic disorders [5,6]. Therefore, identifying natural compounds capable of selectively reprogramming microbial–metabolic trajectories has become an important goal in nutritional intervention and therapeutic development.

Flavonoids, a broad class of polyphenolic compounds widely distributed in functional foods, are promising candidates for such interventions. Because of their low oral bioavailability (<5%), most ingested flavonoids escape absorption in the small intestine and reach the colon intact [7,8]. In this environment, bidirectional interactions occur. Colonic microbes enzymatically deglycosylate and cleave flavonoid structures to generate absorbable and highly bioactive phenolic metabolites. Conversely, parent compounds and their catabolites can selectively suppress opportunistic pathogens while promoting beneficial symbionts [9,10,11]. This axis of microbiota-directed biotransformation coupled with compound-driven ecological remodeling is considered a major mechanism underlying the gastrointestinal protective effects of polyphenols.

Astilbin is a highly bioactive dihydroflavonol rhamnoside widely distributed in functional foods and edible-medicinal plants, with abundant sources including the leaves of *Engelhardtia roxburghiana* and *Lithocarpus polystachyus* (commonly consumed as sweet tea). It possesses well-documented anti-inflammatory, antioxidant, and immunomodulatory properties [12,13,14]. Previous in vivo studies, including our recent work, have demonstrated that dietary astilbin supplementation effectively ameliorates high-fat diet-induced metabolic dysfunction and hepatic lipid accumulation [15]. Given its low systemic absorption, the pharmacological effects of astilbin are likely mediated, at least in part, by the gut microbiota [15,16]. However, most rodent-based in vivo studies have relied primarily on cecal sampling. In humans, microbial flavonoid biotransformation occurs mainly in the colon. Because of substantial anatomical and microbial differences between the murine cecum and the human colon, mouse models may not fully capture human-specific astilbin metabolism. Although limited in vitro studies suggest that gut microbial communities can deglycosylate astilbin to its aglycone form [15,17], the sustained reciprocal interactions between astilbin and complex human microbial consortia remain unclear. Most existing studies rely on endpoint analyses, which cannot resolve the dynamic, multistage biotransformation processes and corresponding microbial successions that unfold over time.

Consequently, the stepwise, time-resolved manner in which the human gut microbiota transforms astilbin remains largely unexplored. To overcome the limitations of traditional endpoint analyses, this study aimed to decipher the high-resolution temporal dynamics of astilbin-associated microbial remodeling. Using a simulated human colonic in vitro model over a continuous 7-day period, we hypothesized that sustained astilbin exposure does not merely exert a static prebiotic effect, but rather is associated with a dynamic, multi-stage ecological succession. By tracking microbiome and metabolome kinetics, we sought to determine how initial microbial cleavage of astilbin is temporally linked to an ecological inflection and how this transient adaptation potentially converges into a putative anti-inflammatory and barrier-protective metabolic state.

## 2. Materials and Methods

### 2.1. Chemicals

Astilbin (≥98% purity) was supplied by Chengdu DeSiTe Biotechnology Co., Ltd., Chengdu, China. Pepsin, gastric lipase, trypsin, pancreatic lipase, porcine bile salts, and corn oil, all of biochemical grade, were purchased from Shanghai Yuanye Bio-Technology Co., Ltd. (Shanghai, China). All other reagents and standards used for metabolomic analyses were of analytical grade (≥98% purity) and obtained from Sigma-Aldrich, St. Louis, MO, USA. The genomic DNA purification kit used for DNA extraction was purchased from Sangon Biotech (Shanghai) Co., Ltd., Shanghai, China.

### 2.2. Participants and Ethics Statement

Three healthy volunteers aged 20–30 years were enrolled. Inclusion criteria included a normal BMI (18.5–24.0 kg/m^2^), regular dietary habits, no history of inflammatory bowel disease and no use of antibiotics or probiotics within the preceding 6 months. A fecal sample (5 g) was collected from each participant for subsequent experiments. This study was approved by the Medical Ethics Committee of Jiangnan University (approval number: JNU20250103IRB16). The study protocol complied with the Measures for the Ethical Review of Biomedical Research Involving Human Beings, the International Ethical Guidelines for Health-related Research Involving Humans, and the Declaration of Helsinki. The research was conducted in accordance with Good Clinical Practice (GCP), the International Council for Harmonisation (ICH) Guideline for Good Clinical Practice, and applicable domestic laws and regulations.

### 2.3. In Vitro Simulated Oro-Gastrointestinal Digestion

In vitro digestion was performed according to the standardized INFOGEST static protocol [17]. Based on our previous in vivo murine study and relevant literature, a single oral dose of astilbin (100 mg) was dissolved in 25 mL of deionized water and used as the test sample. The 100 mg dose of astilbin was extrapolated from our previous in vivo murine study (20 mg/kg BW). According to the FDA human equivalent dose (HED) conversion formula based on body surface area [HED (mg/kg) = animal dose (mg/kg) × (animal Km/human Km)], the equivalent dose for a 60 kg adult human is approximately 97.2 mg (rounded to 100 mg). This dosage reflects a physiologically relevant concentration attainable through the consumption of astilbin-rich functional beverages or dietary supplements.

Simulated salivary fluid (SSF), simulated gastric fluid (SGF), and simulated intestinal fluid (SIF) were prepared according to the protocol. During the oral phase, the sample was mixed with 20 mL of SSF, followed by the addition of CaCl_2_ and H_2_O, and incubated at 37 °C for 2 min with shaking. The resulting oral bolus was mixed with 40 mL of SGF, gastric lipase (60 U/mL), and pepsin (2000 U/mL), and incubated at 37 °C with continuous stirring at 150 rpm. The pH was automatically maintained at 3.0. Subsequently, the gastric digest was combined with 34 mL of SIF, trypsin (100 U/mL), pancreatic lipase (2000 U/mL), and bile salts (10 mM), and incubated under the same temperature and stirring conditions, with the pH automatically maintained at 7.0.

### 2.4. In Vitro Simulated Colonic Fermentation

**Figure 1:**
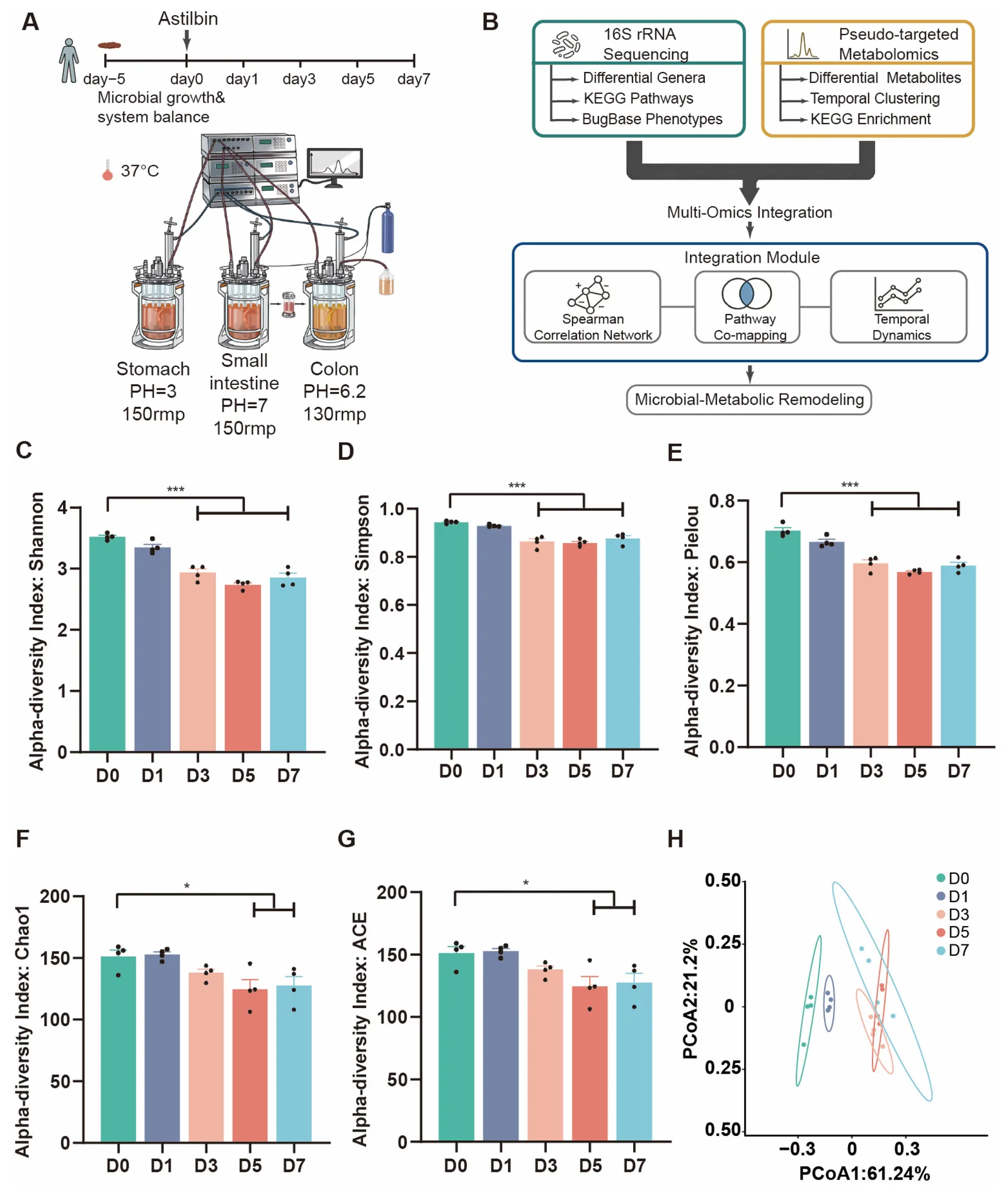
Experimental design, multi-omics pipeline, and temporal trajectories of gut microbial diversity associated with long-term astilbin intervention. (**A**) Schematic representation of the in vitro simulated human gastrointestinal digestion and colonic fermentation model. Following a 5-day stabilization period, the colonic microbiota was continuously exposed to astilbin, with sampling performed at multiple time points (D0, D1, D3, D5, and D7). (**B**) Overview of the multi-omics analytical pipeline, **where arrows indicate the sequence of data processing and integration steps**. (**C**–**E**) Temporal dynamics of microbial community diversity and evenness, assessed by the Shannon (**C**), Simpson (**D**), and Pielou (**E**) indices. (**F**,**G**) Temporal dynamics of microbial community richness, estimated by the Chao1 (**F**) and ACE (**G**) indices. (**H**) Principal Coordinate Analysis (PCoA) plot demonstrating temporal succession of the microbial community structure. Statistical significance in (**C**–**G**) versus D0 was evaluated by one-way ANOVA with Dunnett’s post hoc test (* *p* < 0.05, *** *p* < 0.001).

After small intestinal digestion, the digest was centrifuged at 4000× *g* for 10 min at 4 °C to separate the supernatant soluble fraction from the undigested solid residue (Figure 1A). The basal colonic fermentation medium, containing corn starch, yeast extract, tryptone, inorganic salts, and Tween 80, was autoclaved at 121 °C for 15 min. After cooling, mucin, L-cysteine hydrochloride, hemin, and trace element solution were added aseptically. Fresh fecal samples from the three healthy volunteers were suspended in sterile PBS (pH 6.8) under anaerobic conditions, filtered through three layers of gauze, and used as the inoculum at 10% (*v*/*v*).

Fermentation was performed in a single fermenter with a working volume of 300 mL to simulate colonic conditions. Mucin–agar spherical particles (pH 6.2) were sterilized by ultraviolet irradiation, and five particles were initially introduced into the fermenter to mimic the colonic mucosa. Throughout fermentation, the pH was automatically maintained at 6.2 using 1 mol/L NaOH and 0.5 mol/L HCl, the temperature was maintained at 37 °C, and strict anaerobiosis was ensured by daily nitrogen flushing. To stabilize the gut microbiota, a 5-day pre-fermentation period was conducted from day −5 to day 0. During this period, 300 mL of fresh medium was replaced daily, an equal volume of waste medium was removed, and three mucin particles were renewed.

After stabilization, formal fermentation was conducted for 7 days from D0 to D7 (Figure 1A). Each day, 5 mL of small intestinal supernatant, corresponding to 10% of the total 50 mL digest volume, and the entire undigested solid residue were introduced into the fermenter. Medium replacement, mucin particle renewal, pH control, temperature control, and anaerobic conditions were maintained as described above.

To ensure representative sampling and account for potential spatial heterogeneity despite continuous stirring, multiple intra-vessel technical replicates were collected from different layers (upper, middle, and lower) of the fermenter at each time point (D0, D1, D3, D5, and D7). Specifically, four technical replicates (*n* = 4) per time point were allocated for 16S rRNA gene sequencing, and five technical replicates (*n* = 5) per time point were allocated for metabolomic profiling, where analytical variability is inherently larger. These positional replicates were individually processed and analyzed to capture within-vessel variance.

### 2.5. 16S rRNA Gene Sequencing and Analysis

Total DNA was extracted using a genomic DNA purification kit (Sangon Biotech, Shanghai, China). The V4 region of the bacterial 16S rRNA gene was amplified using primers 515F/806R with Phusion High-Fidelity PCR Master Mix (New England Biolabs, Ipswich, MA, USA) and a 30-cycle amplification program. PCR products were examined by agarose gel electrophoresis, purified using magnetic beads, pooled in equimolar amounts, and recovered from the target bands. After quality control using Qubit and qPCR, paired-end sequencing (PE250) was performed on an Illumina NovaSeq 6000 platform (Illumina Inc., San Diego, CA, USA).

Sequencing data were processed using QIIME 2. Briefly, paired-end reads were first assigned to samples based on their unique barcodes and trimmed to remove barcode and primer sequences. Quality filtering was then performed using fastp (v0.23.1) prior to DADA2-based denoising to generate amplicon sequence variants (ASVs) and a feature table. To correct for uneven sequencing depth across samples, the ASV count table was rarefied to the minimum sample depth observed in the dataset. All subsequent alpha- and beta-diversity analyses were performed on this rarefied dataset to ensure comparability across time points. Taxonomic annotation was performed against the Greengenes database. Functional predictions were conducted using PICRUSt2 (v2.3.0) and Tax4Fun (v0.3.1), and microbial phenotypes were inferred using the BugBase tool (https://bugbase.cs.umn.edu, accessed on 8 June 2026) (Figure 1B).

### 2.6. Metabolite Extraction and Analysis

All sample preparation procedures were performed on ice. Briefly, 100 μL of fermentation broth was mixed with 400 μL of cold methanol containing a mixed internal standard solution. This internal standard mixture, adopted from the standardized pseudo-targeted metabolomics protocol, comprised 14 isotope-labeled and exogenous compounds, including Trp-d5, Phe-d5, and Cholic acid-d4. The complete list of compounds and their final concentrations is detailed in Table S2 [18]. The mixture was vortexed for 2 min and sonicated in an ice-water bath for 15 min. After centrifugation at 12,000 rpm for 20 min at 4 °C, two 200 μL aliquots of the supernatant were transferred to new tubes, lyophilized or vacuum-concentrated, and stored at −80 °C until analysis. Before injection, dried extracts were reconstituted in 100 μL of 20% methanol/water, vortexed, sonicated, and centrifuged. The resulting supernatant was subjected to pseudo-targeted metabolomic analysis using an AB Sciex QTRAP 5500 mass spectrometer (AB Sciex, Framingham, MA, USA) coupled to a UHPLC system equipped with a Waters Acquity BEH C8 column (100 mm × 2.1 mm, 1.7 μm; Waters Corporation, Milford, MA, USA). Data were acquired in both positive and negative ionization modes using dynamic multiple reaction monitoring (dMRM) in accordance with this established protocol. Detailed instrumental parameters are provided in Table S1. Data processing and statistical analysis were performed using MS-DIAL 5.1 and MetaboAnalyst 6.0.

### 2.7. Statistical Analysis

All experimental data are presented as the mean ± standard error (SE). Statistical analyses were performed using R-4.5.3 (R Foundation for Statistical Computing, Vienna, Austria) and SPSS 20.0 (IBM Corp., Armonk, NY, USA).

Given the single pooled fermenter design (*n* = 1 biological replicate), it must be explicitly noted that all variance (mean ± SE) and statistical comparisons across time points evaluate the temporal fluctuations of intra-vessel technical replicates within this specific continuous culture. Accordingly, the statistical significance presented in this study reflects temporal trends and technical stability within this defined microbial system, rather than inter-individual biological variance.

For the longitudinal comparisons of microbial alpha-diversity indices and relative abundances of specific microbial taxa, a one-way analysis of variance (ANOVA) was performed, followed by Dunnett’s post hoc test to account for multiple comparisons specifically when comparing successive time points (D1, D3, D5, and D7) against the baseline (D0). For metabolomic data, the False Discovery Rate (FDR, Benjamini–Hochberg procedure) was applied for multiple comparison corrections to control false positives. A probability value of *p* < 0.05 (or adjusted *p* < 0.05) was considered statistically significant.

## 3. Results

### 3.1. Temporal Restructuring of the Colonic Microbial Community During Astilbin Exposure

To evaluate the ecological effects of astilbin, we first tracked dynamic changes in microbial diversity and community structure. Alpha diversity metrics, including the Chao1, Shannon, and Simpson indices, showed a highly consistent kinetic pattern. Diversity initially declined during the early fermentation stages (D1–D5), suggesting selective loss of astilbin-sensitive taxa, followed by a modest rebound at D7 (Figure 1C–G). These results suggest that sustained astilbin exposure is associated with a microbial community trajectory characterized by an initial selection bottleneck, followed by a shift toward a newly re-equilibrated state. Principal coordinate analysis (PCoA) further supported this pattern, showing a directional and stepwise temporal succession from the D0 baseline, through an intermediate transitional phase (D3–D5), to the final D7 endpoint (Figure 1H).

**Figure 2:**
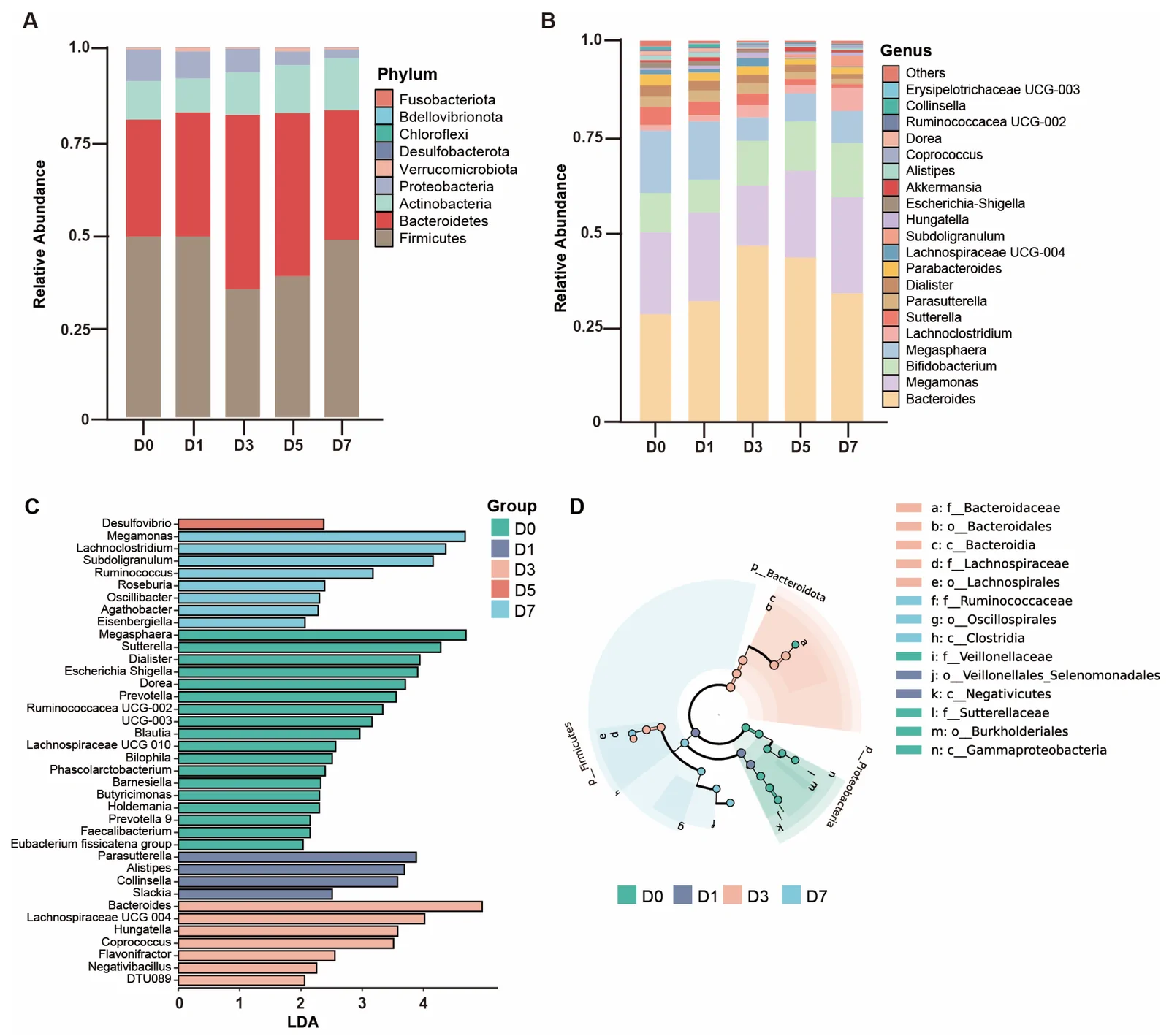
Astilbin correlates with profound taxonomic restructuring and sequential enrichment of stage-specific microbial biomarkers. (**A**,**B**) Temporal shifts in the relative abundance of the colonic microbial community at the phylum (**A**) and genus (**B**) levels. Only the most abundant taxa are shown. (**C**) Linear discriminant analysis (LDA) scores identifying biomarker genera significantly enriched at specific temporal phases (LDA score > 2.0, *p* < 0.05). (**D**) Cladogram representing the phylogenetic architecture and distribution of the differentially enriched taxa.

Taxonomic profiling and LEfSe analysis further identified the microbial taxa contributing to this succession (Figure 2A,B). The early baseline stage (D0–D1) was characterized by a high abundance of opportunistic pathogens, including *Escherichia-Shigella* and *Bilophila*. By D3, a critical functional transition had occurred, marked by rapid enrichment of polysaccharide-degrading and polyphenol-metabolizing specialists, particularly *Bacteroides* and *Flavonifractor* (LDA = 4.96). Prolonged exposure until D7 consolidated a beneficial anaerobe-enriched community dominated by key butyrate producers and strict anaerobes, including *Megamonas*, *Subdoligranulum*, *Ruminococcus*, and *Roseburia*. Together, these structural shifts indicate that prolonged astilbin exposure is temporally associated with a progressive decrease in opportunistic pathogens and a concurrent enrichment of a fiber-degrading, SCFA-producing consortium (Figure 2C,D).

### 3.2. Functional Prediction Suggests Extensive Shifts in Functional Potential and Phenotypes

**Figure 3:**
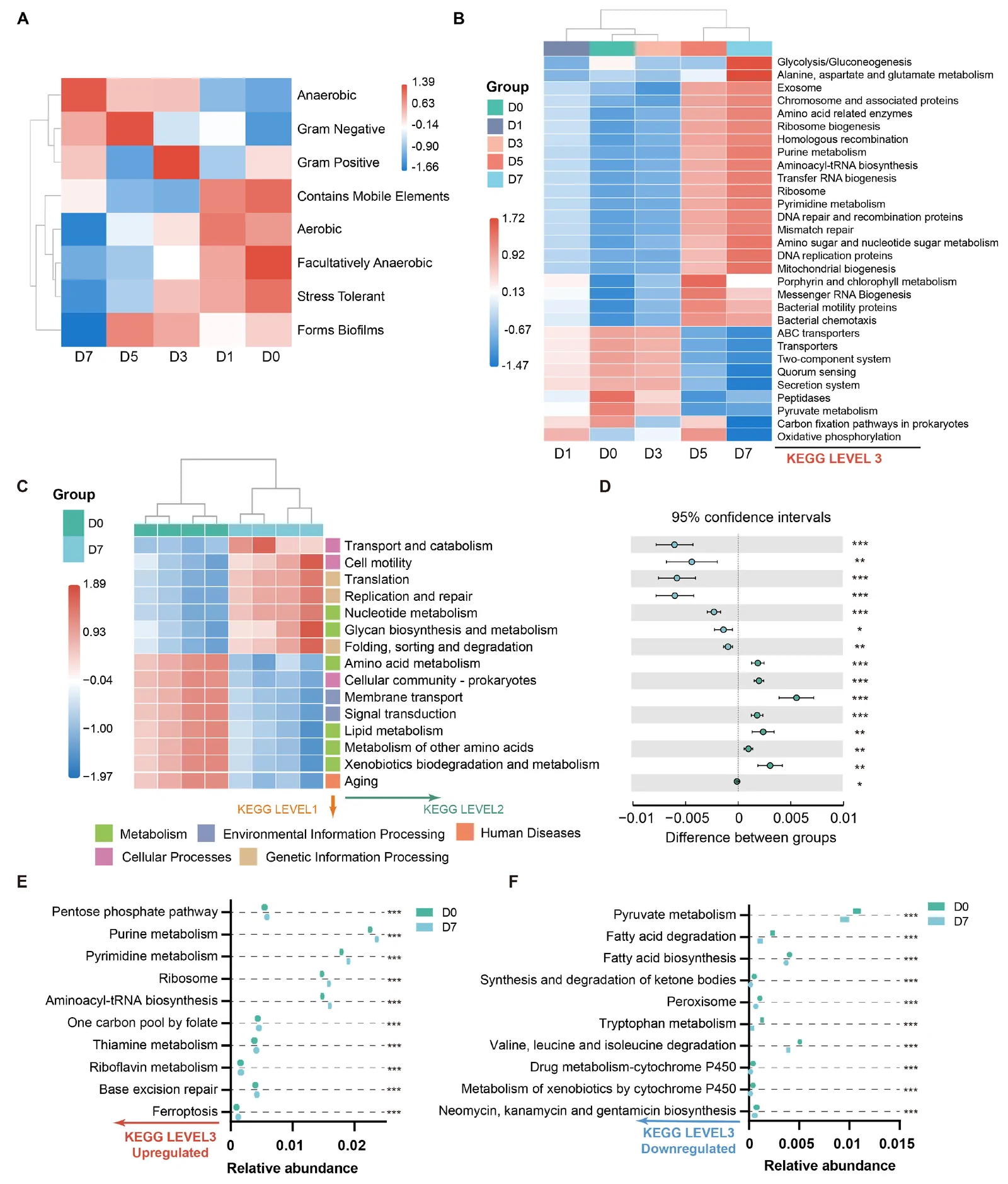
Predictive functional profiling reveals phenotypic optimization and global functional potential remodeling of the gut microbiota associated with astilbin. (**A**) Temporal dynamics of community-level phenotypes predicted by BugBase. (**B**) Global overview of the top significantly altered KEGG Level 3 functional pathways predicted by PICRUSt2. (**C**) Hierarchical clustering heatmap of differential KEGG Level 2 pathways between D0 and D7. (**D**) Extended error bar plot showing significant differences in mean proportions corresponding to panel (**C**), with 95% confidence intervals displayed. (**E**,**F**) Significantly up-regulated. (**E**) and down-regulated (**F**) predicted KEGG Level 3 metabolic pathways at D7 relative to D0 (* *p* < 0.05, ** *p* < 0.01, *** *p* < 0.001).

To translate these taxonomic shifts into ecological traits, community-level phenotypes were inferred using BugBase (Figure 3A). Astilbin intervention progressively enriched the obligately anaerobic phenotype, which peaked at D7. In contrast, dysbiosis-associated phenotypes, including Potentially Pathogenic, Stress Tolerant, and Aerobic phenotypes, showed a continuous decline throughout fermentation. These phenotypic shifts suggest that astilbin exposure is associated with favorable shifts in the colonic microenvironment and a reduction in conditions favorable for aerobic opportunistic pathogens.

PICRUSt2 was then used to infer the functional potential remodeling underlying this phenotypic optimization (Figure 3B–F). Comparison of global functional profiles, particularly between the D0 baseline and D7 endpoint, revealed a substantial shift in microbial energy allocation. Pathways associated with cellular proliferation, genetic fidelity, and biosynthesis, including translation, DNA repair, purine metabolism, and the pentose phosphate pathway, were markedly upregulated, suggesting a shift toward a state with higher predicted metabolic activity and biosynthesis potential.

In contrast, pathways associated with environmental stress and toxicity were strongly depleted. Significant downregulation of valine, leucine and isoleucine degradation and Tryptophan metabolism, which are commonly associated with harmful deamination processes, suggested a predicted reduction in pathways associated with amino acid degradation. In addition, depletion of fatty acid degradation, Peroxisome, and metabolism of xenobiotics by cytochrome P450 suggested reduced oxidative stress burden and decreased detoxification demand. Collectively, these predicted functional shifts indicate that astilbin exposure is associated with a redirection of the gut microbiota from a stress-defensive and proteolytic state toward a metabolic regime dominated by biosynthesis and homeostasis.

### 3.3. Emergence of a Homeostatic Metabolic Signature Following Long-Term Astilbin Intervention

**Figure 4:**
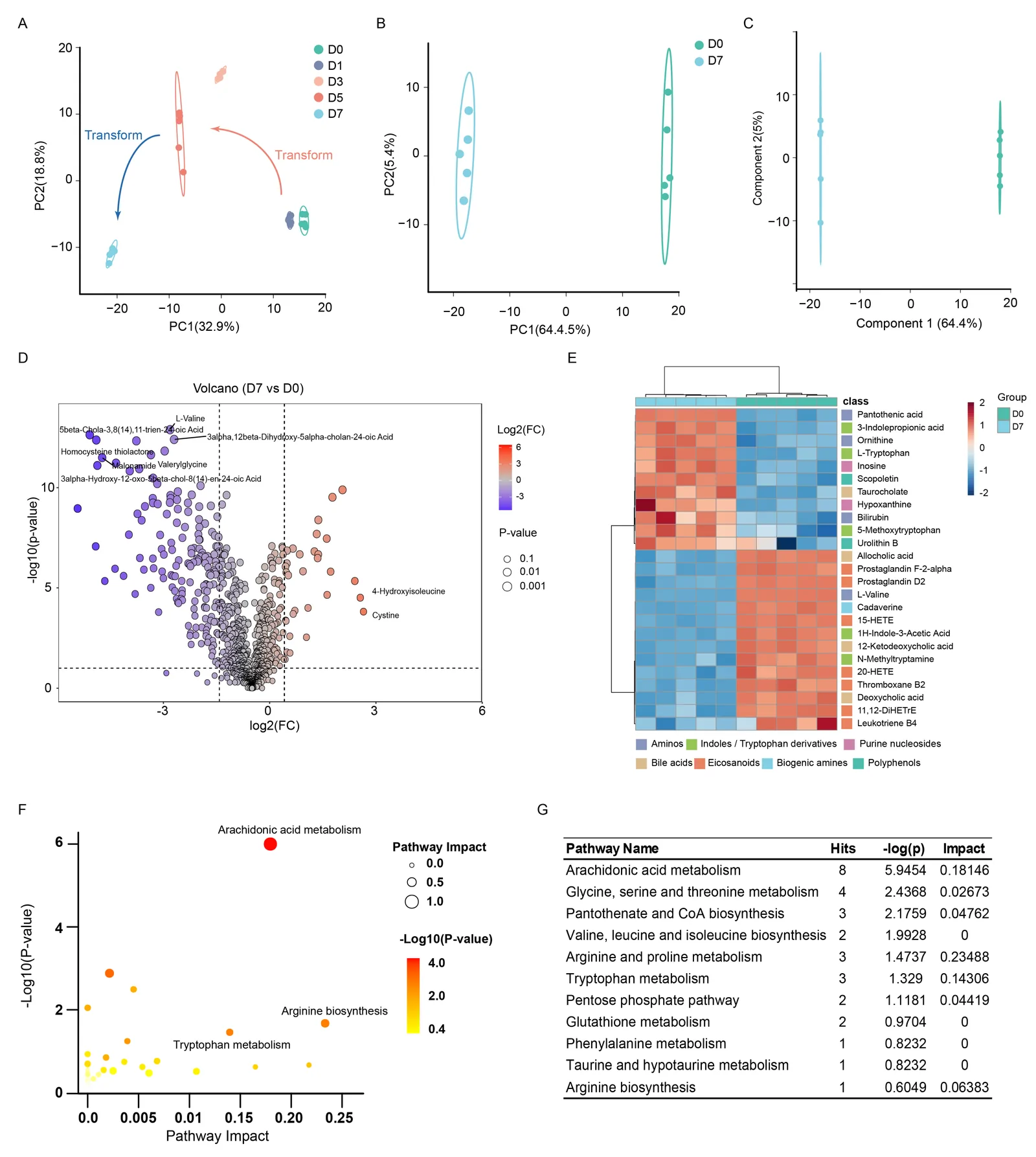
Pseudo-targeted metabolomics profiling reveals astilbin-associated temporal metabolic trajectories and terminal chemical reprogramming. (**A**) Principal component analysis (PCA) score plot across all five time points. (**B**,**C**) Pairwise PCA (**B**) and OPLS-DA (**C**) score plots comparing D0 and D7. (**D**) Volcano plot of differential metabolites (Variable Importance in Projection (VIP) > 1.0, adjusted *p* (FDR) < 0.05, |log_2_ Fold Change (FC)| > 1.0). Dashed lines indicate the significance thresholds of adjusted *p* < 0.05 and |log2 FC| > 1.0. (**E**) Heatmap of representative differential metabolites annotated by biochemical class (left color bar). Color intensities indicate z-score normalized abundances. (**F**,**G**) KEGG pathway enrichment analysis. Node color and size reflect -log10(*p*-value) and pathway impact, respectively. (Abbreviations: 3-IPA, 3-indolepropionic acid; LTB4, leukotriene B4; TXB2, thromboxane B2; PGD2, prostaglandin D2).

To validate the 16S rRNA gene-based functional predictions, pseudo-targeted metabolomic profiling was performed across the entire fermentation timeline. Principal component analysis (PCA) revealed clear temporal evolution, with metabolomic profiles naturally segregating into three distinct kinetic phases: D0–D1, D3–D5, and D7 (Figure 4A). Orthogonal partial least squares discriminant analysis (OPLS-DA) comparing the D0 baseline with the D7 endpoint confirmed a robust metabolic shift (R^2^X = 0.685, R^2^Y = 1.000, Q^2^ = 0.930), and 100-permutation testing indicated no overfitting (Figure 4B,C). Using VIP > 1, adjusted *p* (FDR) < 0.05, and |log_2_(FC)| > 1 as screening criteria, 196 differential metabolites were identified, including 165 downregulated and 31 upregulated metabolites (Figure 4D). Notably, barrier-protective metabolites, such as indole-3-propionic acid (3-IPA) and pantothenic acid, were highly enriched, whereas pro-inflammatory mediators, including Leukotriene B4 (LTB4) and Thromboxane B2 (TXB2), and putrefaction markers, such as 2-methylbutyric acid, were strongly depleted (Figure 4E). Pathway enrichment analysis further confirmed significant perturbations in arachidonic acid and tryptophan metabolism (Figure 4F,G), indicating comprehensive functional potential remodeling.

To characterize the temporal evolution of these core metabolites, their abundance trajectories were mapped across all five time points (Figure S1A–L). In the tryptophan metabolism module, 3-IPA and its precursor L-tryptophan increased continuously, whereas indole-3-acetic acid (IAA) declined steadily. Although IAA can act as a weak local aryl hydrocarbon receptor (AhR) agonist, its systemic accumulation is strongly associated with protein putrefaction and is clinically recognized as a pro-inflammatory uremic toxin linked to indoxyl sulfate production. Thus, the continuous decline in IAA, together with the marked accumulation of 3-IPA, reflects a potential microbial–metabolic diversion away from potentially toxic proteolytic pathways and toward putative protective indole biosynthesis.

Markers of protein proteolytic fermentation, including L-valine and cadaverine, were progressively depleted. Notably, key pro-inflammatory lipid mediators, including LTB4, Prostaglandin D2 (PGD2), and TXB2, shared a characteristic biphasic kinetic pattern. These metabolites peaked sharply at D3 and then declined to near-baseline levels by D7, capturing a complete burst-and-resolution trajectory of the arachidonic acid cascade. Collectively, these distinct kinetic patterns, including sustained accumulation, progressive depletion, and biphasic resolution, reinforce D3 as a highly dynamic transitional phase and highlight that astilbin exposure is statistically associated with a potential shift toward a durable anti-inflammatory homeostatic state.

### 3.4. Temporal Metabolomic Profiling Reveals a Highly Dynamic Transitional Phase and Distinct Metabolic Trajectories

**Figure 5:**
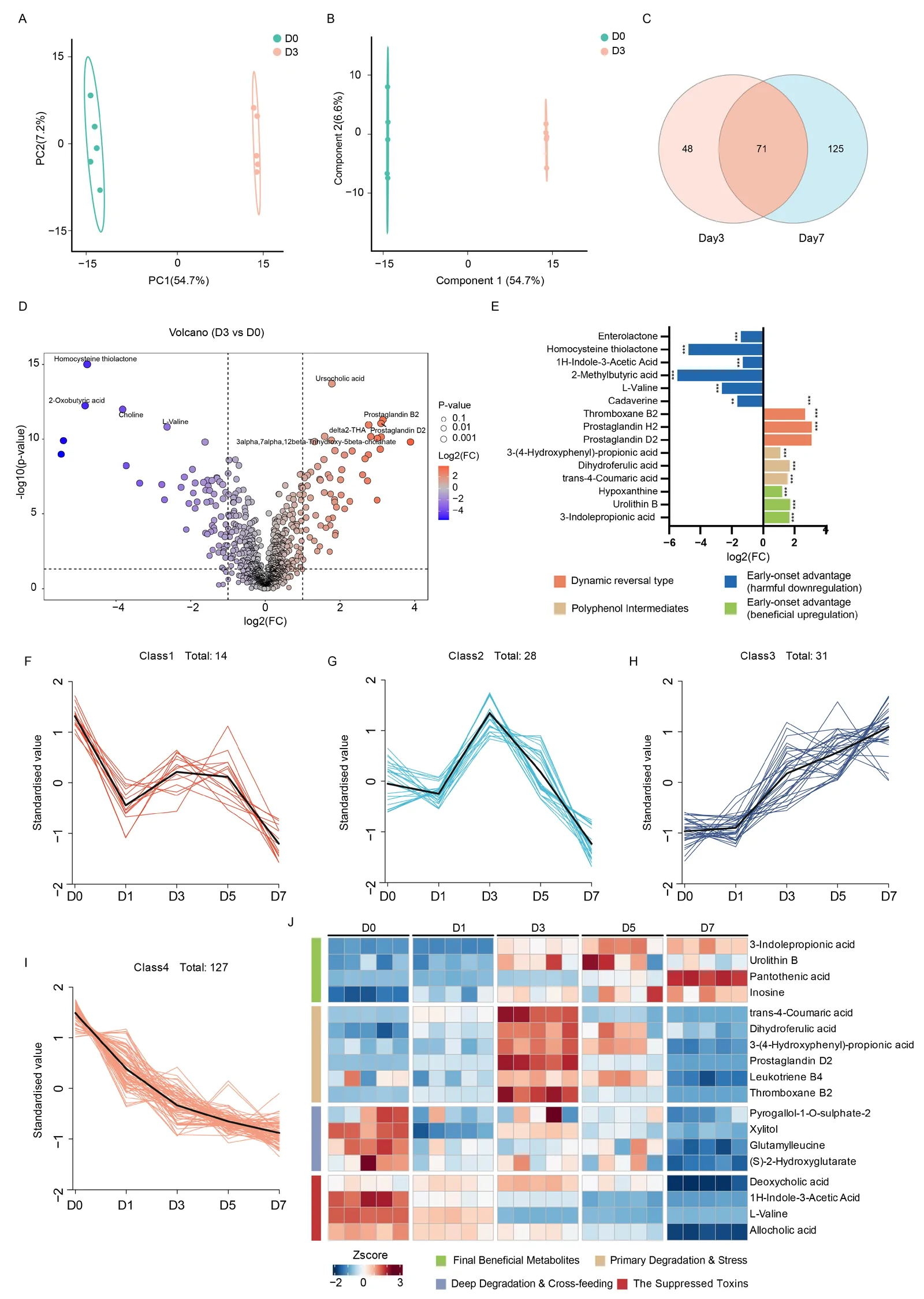
Time-series metabolomic tracking identifies Day 3 as a critical inflection point and unveils distinct kinetic trajectories of functional potential remodeling. (**A**,**B**) PCA (**A**) and OPLS-DA (**B**) score plots comparing D0 and D3. (**C**) Venn diagram illustrating the intersection of differential metabolites between D3 (vs. D0) and D7 (vs. D0). (**D**) Volcano plot showing significantly differential metabolites at D3 compared to D0. (**E**) Log_2_(FC) of representative core metabolites at D3, categorized by functional role to illustrate the dual signal of protective outputs and transient stress (** *p* < 0.01, *** *p* < 0.001). (**F**–**I**) Temporal tracking of significant differential metabolites using K-means clustering, revealing four distinct kinetic patterns (Classes 1–4) across the 7-day period. In panels (**F**–**I**), thin colored lines represent the kinetic trajectories of individual metabolites within each cluster, while the thick black lines indicate the mean trajectory (cluster center). (**J**) Temporal heatmap of key representative metabolites annotated by ecological module. (Abbreviations: IAA, indole-3-acetic acid; DCA, deoxycholic acid).

To determine how the terminal homeostatic signature was established, we further analyzed metabolomic changes across all time points. PCA revealed clear temporal segregation into three phases, D0–D1, D3–D5, and D7, suggesting D3 as a potential critical transitional node (Figure 5A). Comparison between D0 and D3 identified 118 differential metabolites, of which 71 persisted to the D7 endpoint (Figure 5B–D), suggesting that metabolic remodeling was initiated early during fermentation.

Biologically, the D3 metabolome displayed a distinct dual signature characterized by early stress and the initiation of homeostasis (Figure 5E). Reduction in specific markers of proteolytic fermentation was already evident at D3, as reflected by decreased cadaverine and IAA levels, whereas protective metabolites, including 3-IPA, urolithin B, and inosine, had begun to accumulate. In contrast to their overall decline at D7, pro-inflammatory lipid mediators, including PGD2 and TXB2, and multiple oxidized bile acids increased significantly at D3. In addition, transient accumulation of trans-4-coumaric acid and dihydroferulic acid confirmed active microbial ring cleavage of the astilbin backbone. This burst-and-resolution pattern suggests that D3 represents a highly dynamic stage characterized by active polyphenol degradation and pronounced biochemical remodeling.

To clarify how the system transitioned from this stress-associated peak to final homeostasis, the D3 and D7 metabolomes were compared (Figure S2A–D). This phase was characterized by extensive metabolic clearance. Pro-inflammatory lipids and oxidized bile acids that increased at D3 were markedly depleted by D7, while early harmful metabolites, including IAA and cadaverine, remained persistently depleted (Figure S2E). At the same time, metabolites initially depleted, such as pantothenic acid and succinate, re-accumulated robustly by D7, suggesting restoration of vitamin biosynthesis and carbohydrate fermentation. The marked elevation of cystine further indicated a shift from oxidative depletion toward restored antioxidant capacity, completing the transition to a durable eubiotic state.

To further characterize dynamic functional potential remodeling during in vitro fermentation, time-series K-means clustering was performed across all time points. Rather than following a simple linear shift, the metabolome exhibited highly coordinated temporal trajectories. As shown in Figure 5F–I, differential metabolites were grouped into four major clusters based on abundance dynamics, revealing a multistage biotransformation process. Cluster 2 showed a biphasic inverted-V trajectory, characterized by a rapid increase at D3 followed by a sharp decline at D7. A temporal heatmap of representative metabolites (Figure 5J) further clarified the biological significance of this phase. Polyphenol degradation intermediates, including trans-4-coumaric acid and dihydroferulic acid, accumulated transiently, indicating active early-stage cleavage of astilbin. Concurrently, pro-inflammatory lipid mediators, including PGD2 and LTB4, showed a pronounced but transient increase at D3. These inflammatory markers were largely cleared by D7, indicating a transition from an initial stress response to marked resolution of inflammatory signatures.

Following this initial metabolic perturbation, the fermentation system evolved toward stable homeostasis. Cluster 3, comprising metabolites that continuously increased over time, was enriched in barrier-protective and beneficial microbial outputs. Representative metabolites, including 3-IPA, succinate, and pantothenic acid, accumulated robustly through D7. In contrast, Cluster 4 represented a continuous depletion trajectory. Toxic secondary bile acids, such as deoxycholic acid (DCA), and proteolytic byproducts, including IAA and L-valine, progressively declined to baseline levels. Together, these temporal dynamics demonstrate that astilbin exposure is statistically linked to a restructuring of the metabolic environment, characterized by the resolution of early inflammatory bursts and the emergence of a durable, 3-IPA-associated homeostatic state.

### 3.5. Temporal Dynamics of the Gut Microbiota Reveal Distinct Ecological Roles Accompanying the Metabolic Inflection Point

**Figure 6:**
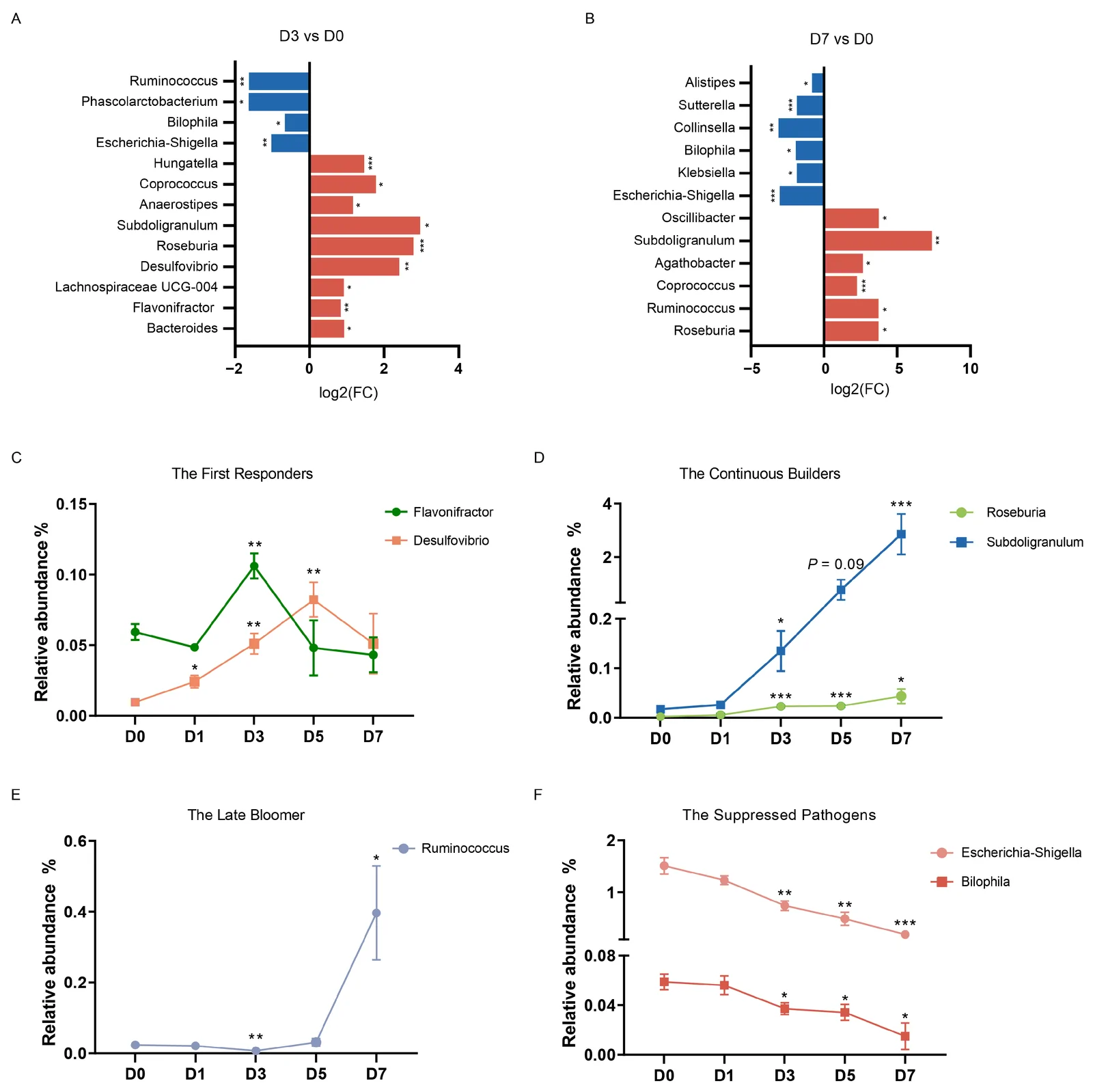
Sequential enrichment and displacement of stage-specific keystone taxa correlate with the dynamic ecological succession. (**A**,**B**) Log_2_(FC) of significantly differential genera at D3 (**A**) and D7 (**B**) relative to D0. Red bars indicate significantly enriched taxa, whereas blue bars represent significantly depleted taxa. (**C**–**F**) Temporal kinetic trajectories of relative abundance for core representative genera across the 7-day fermentation period, conceptually categorized into four functional guilds: (**C**) The First Responders, peaking transiently at D3; (**D**) The Continuous Builders, expanding steadily from D3 through D7; (**E**) The Late Bloomers, showing delayed enrichment at D7; and (**F**) The Suppressed Pathogens, progressively depleted throughout the intervention. Statistical significance for specific taxa versus D0 was evaluated using one-way ANOVA with Dunnett’s post hoc test (* *p* < 0.05, ** *p* < 0.01, *** *p* < 0.001).

To determine whether these metabolic trajectories were associated with coordinated microbial succession, temporal 16S rRNA gene profiles were integrated with the K-means clustering framework. This analysis identified four distinct microbial ecological roles that sequentially characterized the fermentation process (Figure 6A,B).

First, the “First Responders”, including *Flavonifractor* and *Hungatella*, mirrored the transient metabolic surge observed in Cluster 2. Notably, *Flavonifractor*, a specialized flavonoid degrader, peaked precisely at D3, consistent with the transient accumulation of polyphenol intermediates. These taxa acted as metabolic pioneers by initiating astilbin cleavage before declining by D7 (Figure 6C).

Following this initial cleavage, the “Continuous Builders,” including *Roseburia*, *Subdoligranulum*, and *Coprococcus*, emerged at D3 and expanded robustly through D7. As major butyrate producers, these taxa were statistically associated with the sustained accumulation of potentially protective metabolites, including 3-IPA and succinate, observed in Cluster 3 (Figure 6D). A third group, the “Late Bloomers” represented by *Ruminococcus*, showed a distinct suppressed-then-recovered pattern. Their late enrichment at D7 coincided with the re-accumulation of pantothenic acid and succinate, suggesting that they may rely on prior colonizers to complete a mature cross-feeding network (Figure 6E).

In contrast, the “Suppressed Pathogens” including *Escherichia-Shigella*, *Klebsiella*, and *Bilophila*, were progressively depleted from D3 to D7, paralleling the continuous decline of metabolites in Cluster 4. Their gradual elimination was closely associated with the clearance of pro-inflammatory lipids, toxic bile acids, and putrefaction markers.

Together, sequential activation of these functional guilds provides an associative framework linking pioneer cleavage to late-stage cross-feeding and pathogen exclusion, potentially explaining the dynamic nature of the D3 transition and the establishment of the D7 homeostatic state. This coordinated succession underscores the temporal association between astilbin exposure and stage-specific ecological remodeling in the gut microbiota.

### 3.6. Multi-Omics Integration Reveals a Coordinated Microbial–Metabolic Network Underlying Ecological Remodeling

**Figure 7:**
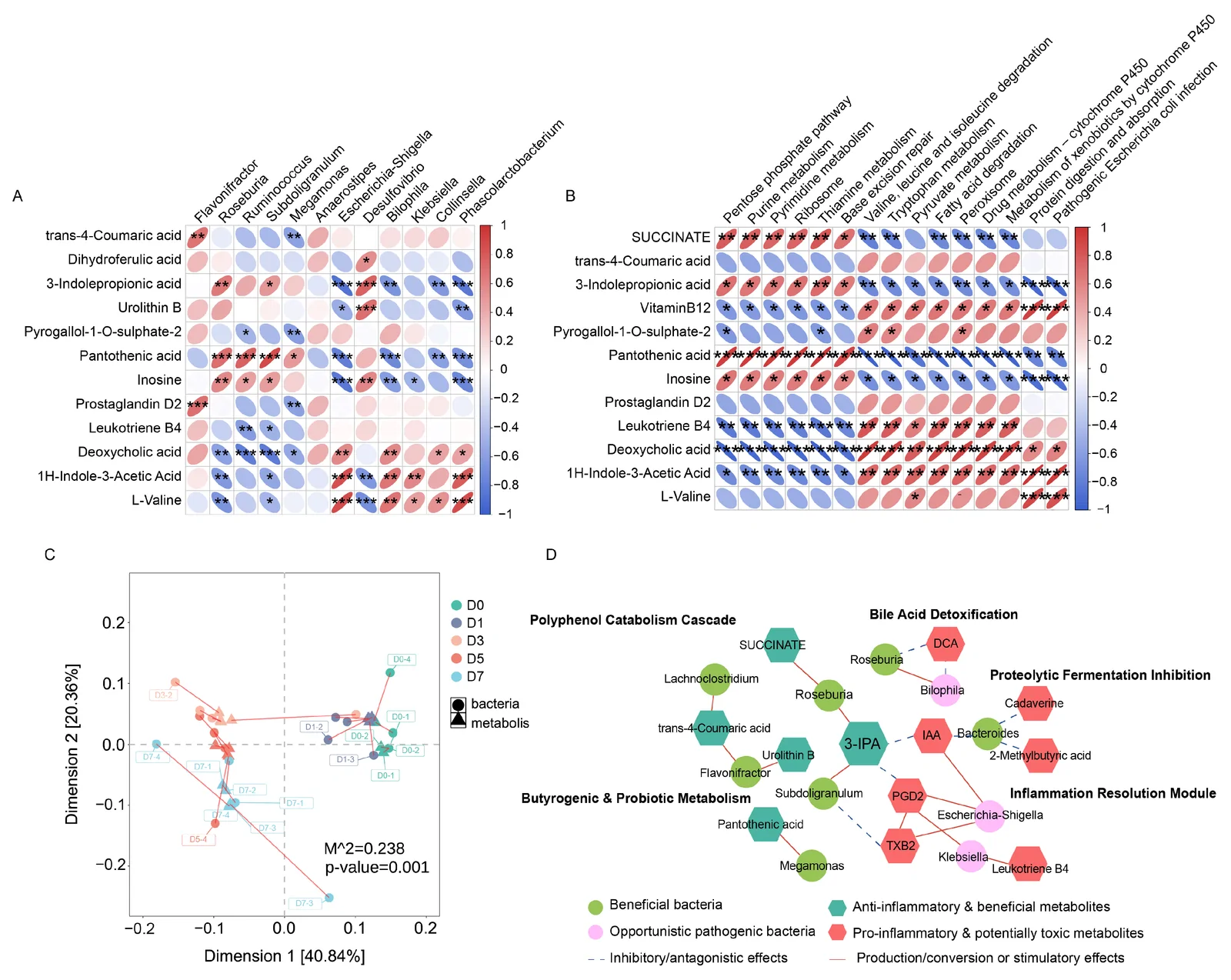
Multi-omics integration reveals a highly coordinated microbial–metabolic statistical interaction network underlying astilbin-associated ecological remodeling. (**A**) Spearman correlation heatmap illustrating associations between stage-specific keystone genera and core differential metabolites. (**B**) Correlations between core metabolites and PICRUSt2-predicted functional pathways. In panels (**A**,**B**), the shape of the ellipse reflects the magnitude of the correlation (narrower ellipses indicate stronger correlations), with right-leaning (red) and left-leaning (blue) ellipses representing positive and negative correlations, respectively (* *p* < 0.05, ** *p* < 0.01, *** *p* < 0.001). (**C**) Global Procrustes analysis evaluating macroscopic temporal concordance between the 16S rRNA microbiome and metabolome. (**D**) Co-occurrence network of key bacteria (circles) and metabolites (hexagons), conceptually partitioned into five ecological modules.

To assess whether temporal functional potential remodeling was associated with microbial succession, global Procrustes analysis was performed by integrating the 16S rRNA gene sequencing and metabolomic datasets (Figure 7C). The analysis revealed significant macro-scale concordance (M^2^ = 0.238, *p* = 0.001), demonstrating that temporal trajectories of the colonic metabolome were closely synchronized with structural shifts in the microbiota.

To further validate whether microbial functional potential was translated into chemical outputs, predicted functional pathways were mapped against the metabolomic profiles (Figure 7B). Beneficial metabolic pathways, including purine and thiamine metabolism, showed strong positive correlations with protective metabolites such as 3-IPA, succinate, and pantothenic acid. Conversely, proteolytic pathways, including valine, leucine, and isoleucine degradation, were positively correlated with proteolytic byproducts, including IAA and L-valine, as well as pro-inflammatory lipids. These results support a strong link between microbial functional potential and observed metabolic output.

At the micro-ecological level, Spearman correlation analysis revealed a highly polarized bipartite interaction network between key taxa and core metabolites (Figure 7A). The pioneer degrader *Flavonifractor* showed strong positive correlations with the polyphenol intermediate trans-4-coumaric acid and the early-stress lipid PGD2, supporting its role in the D3 cleavage event. In contrast, a reciprocal association emerged between late-stage beneficial colonizers and suppressed pathogens. The “Continuous Builders”, including *Roseburia*, *Subdoligranulum*, and *Megamonas*, formed a dense positive correlation cluster with protective terminal metabolites, such as 3-IPA and pantothenic acid, while showing strong negative correlations with toxic substrates. Conversely, opportunistic pathogens, including *Escherichia-Shigella*, *Klebsiella*, and *Bilophila*, were positively correlated with pro-inflammatory lipid mediators, such as LTB4, and secondary bile acid toxicity, represented by DCA, but negatively correlated with 3-IPA.

To synthesize these statistical associations, a multi-omics co-occurrence network was constructed (Figure 7D). Although these correlations do not establish causality, this exploratory network, based on statistical associations, suggests five interconnected domains that may be linked to astilbin exposure: (1) the polyphenol catabolism cascade statistically linked to *Flavonifractor*; (2) the putative beneficial metabolism hub centered on 3-IPA and succinate; (3) the inflammation resolution module, in which suppression of *Escherichia-Shigella* is associated with attenuation of arachidonic acid derivatives; (4) bile acid detoxification driven by *Bilophila* depletion; and (5) inhibition of proteolytic fermentation. However, it must be emphasized that this multi-omics network is built on statistical Spearman correlations. Rather than establishing definitive causal pathways, these modules represent associative patterns. Thus, this integrated network serves as a predictive framework, illustrating how astilbin exposure is potentially associated with the coordination of stage-specific microbial succession to dismantle inflammatory and putrefactive cascades, ultimately contributing to a 3-IPA-dominated homeostatic state. Future orthogonal validation via metagenomic sequencing and in vivo causal modeling is required.

## 4. Discussion

The bidirectional interaction between flavonoids and the gut microbiota is widely recognized as a central mechanism underlying their gastrointestinal protective effects [19,20]. However, the temporal dynamics of these interactions, particularly the sequential order in which they unfold, have rarely been systematically resolved. Unlike conventional studies based on endpoint comparisons, our time-series sampling strategy captured the trajectory of microbial restructuring and functional potential remodeling under sustained astilbin exposure. Our results show that astilbin does not elicit a simple linear prebiotic response. Instead, astilbin exposure is closely associated with a coordinated three-stage ecological succession: an early stress-response phase (D0–D1), a highly dynamic transitional phase (D3), and a late homeostatic state (D5–D7). These temporal data support the view that astilbin acts as a dynamic ecological modulator, progressively shifting the microbial–metabolic regime from proteolytic fermentation and oxidative stress toward carbohydrate fermentation and antioxidant defense.

A key finding of this study was the identification of D3, which may represent a critical metabolic transition point. At this stage, the system exhibited a distinct dual signature: the potential initiation of homeostasis and the emergence of transient chemical stress. On one hand, the enrichment of pioneer taxa such as *Flavonifractor* and *Bacteroides* was temporally correlated with the appearance of astilbin ring-cleavage intermediates (such as trans-4-coumaric acid) and the suppression of proteolysis-related markers. On the other hand, D3 was characterized by a marked increase in pro-inflammatory lipid mediators, including PGD2 (9-fold peak), and oxidized bile acids. This observation is striking yet perplexing. Several alternative explanations should be considered. First, this lipid burst may represent an acute microbial stress response to the initial high concentration of astilbin, rather than a beneficial adaptive remodeling process. Second, it could arise from the non-enzymatic auto-oxidation of lipid precursors present in the basal medium or the simulated digestion fluids. Third, residual enzymatic activities in the fecal inoculum could also contribute. Whether this transient lipid burst occurs in vivo remains highly uncertain, as a healthy host immune system would likely rapidly clear these mediators. Future studies using heat-inactivated inoculum controls or targeted lipidomics of the basal medium would be necessary to pinpoint the exact origin of this phenomenon. Nevertheless, the subsequent comprehensive clearance of these signals by D7 coincided with the expansion of the butyrate-producing consortium, suggesting a potential transition toward an anti-inflammatory state [21].

This metabolic resolution was associated with a potential functional division of labor within the microbiota, which we conceptually categorized into four ecological guilds. The putative “first responders”, such as *Flavonifractor*, may act as metabolic pioneers, contributing to alterations in the initial chemical environment [22]. Their early peak was statistically followed by the “continuous builders”, including *Roseburia*, *Subdoligranulum*, and *Coprococcus*, which consistently correlated with homeostasis-associated SCFA and indole production [23]. The “late bloomers”, including *Megasphaera* and *Ruminococcus*, colonized successfully only after earlier guilds had established, suggesting the potential sequential enrichment of complementary functional guilds. Conversely, “suppressed pathogens”, such as *Escherichia-Shigella* and *Klebsiella*, were progressively outcompeted. This sequential enrichment of complementary symbionts, combined with the exclusion of opportunistic pathogens, is consistent with ecological succession driven by environmental niche modification [24].

Through this microbial succession, the colonic metabolome underwent a profound predictive reprogramming along two interconnected major axes. The first was the barrier protection and detoxification axis. The microbiota profile observed at D7 was predicted to exhibit a redirection of tryptophan metabolism. While IAA can act as a beneficial local aryl hydrocarbon receptor (AhR) agonist, its excessive generation is often linked to proteolytic fermentation [25]. Notably, our data showed a strong statistical correlation with a metabolic diversion away from IAA production and toward the enhanced synthesis of the robustly protective metabolite 3-IPA [26]. In the host context, 3-IPA can activate pregnane X receptor (PXR), thereby strengthening mucosal tight junctions and supporting barrier integrity [27]. Concurrently, the competitive exclusion of Bilophila and other opportunistic pathogens reduced the production of potentially toxic deoxycholic acid (DCA) and proteolytic byproducts such as cadaverine and L-valine [28,29], contributing to detoxification of the microenvironment [30].

The second was the biotransformation and nutritional optimization axis. The Flavonifractor-associated catabolic cascade was statistically linked to the eventual accumulation of urolithin B, a terminal metabolite with enhanced predicted antioxidant potential [31]. Although direct enzymatic evidence was not obtained in this study, the transient accumulation of phenolic acid intermediates, particularly trans-4-coumaric acid and dihydroferulic acid, is consistent with the canonical flavonoid degradation pathway, in which the parent compound is presumed to undergo sequential deglycosylation, C-ring fission, and further phenolic acid metabolism. These intermediates were detected predominantly at D3, coinciding with the peak relative abundance of *Flavonifractor*, a genus known to harbor flavonoid-cleaving enzymes. This temporal correlation supports the hypothesis that astilbin was actively degraded by pioneer taxa prior to terminal biotransformation. However, the specific enzymatic machinery (e.g., rhamnosidases, ring-cleavage dioxygenases) and the precise identity of all intermediate metabolites remain to be confirmed by future targeted enzymatic assays and stable isotope tracing studies. In parallel, the inferred enhancement of microbial purine and vitamin-related metabolic pathways was statistically associated with the observed accumulation of inosine, hypoxanthine, and pantothenic acid (vitamin B5), which may provide micronutrients and energetic substrates that support mucosal repair [32].

An additional noteworthy observation was the V-shaped kinetic trajectory of succinate. Although its D7 concentration did not significantly exceed baseline, succinate showed strong positive Spearman correlations with anabolic pathways, including the pentose phosphate pathway and purine metabolism, and negative correlations with catabolic and stress-related modules. This pattern suggests that dynamic turnover of the succinate pool may reflect reprogramming of microbial carbon flux from catabolism toward anabolism. Succinate is therefore hypothesized to function as a putative cross-feeding currency, although this correlation-based interpretation requires targeted in vivo validation and stable isotope tracing in future studies [33].

In the broader context of polyphenol–microbiota interactions, our findings provide an important temporal dimension. Structurally, astilbin is a dihydroflavonol rhamnoside, which distinguishes it from extensively studied flavonol aglycones such as quercetin and their corresponding glycosides. While many simpler flavonoids undergo relatively rapid microbial degradation [34], the specific dihydroflavonol backbone and rhamnose moiety of astilbin may impose distinct kinetic constraints on microbial cleavage. Consequently, unlike conventional carbohydrate-based prebiotics that are frequently reported to induce a relatively linear expansion of *Bifidobacterium* or *Lactobacillus* [35], astilbin exposure is associated with a highly dynamic, temporally coordinated remodeling. This multi-stage succession is characterized by the sequential enrichment of early flavonoid degraders during an early polyphenol-cleavage phase (D3), followed by the delayed establishment of a secondary butyrogenic consortium (*Roseburia*, *Subdoligranulum*). Previous endpoint-based in vivo studies established the efficacy of astilbin in alleviating metabolic disorders, but the mechanisms driving microbial community shifts remained largely unresolved [15]. Our in vitro time-series analysis helps clarify this process by showing that microbial astilbin metabolism, represented by the cleavage cascade, and compound-driven community remodeling, represented by ecological succession, intersect temporally and functionally at D3.

While this exploratory study provides a high-resolution temporal framework, several methodological limitations should be considered. First, the reliance on a single pooled continuous culture without a parallel vehicle-only control makes it difficult to distinguish astilbin-specific effects from natural microbial adaptation to the basal medium. Second, although pooling establishes a unified baseline, it inevitably masks donor-dependent variation and strain-specific astilbin metabolism [16]. Third, the functional shifts reported here are based on computational predictions rather than direct transcriptomic or biochemical evidence. Finally, because this in vitro system lacks host immune and absorptive mechanisms, the 7-day successional trajectories may not fully reflect chronic in vivo remodeling. Given these constraints, our findings should be regarded as an associative, hypothesis-generating foundation. Future studies employing individual-specific in vivo models, together with shotgun metagenomics and targeted causal validation (such as purified 3-IPA supplementation), will be essential to confirm astilbin’s role as an ecological modulator.

## 5. Conclusions

This exploratory in vitro study, based on a single pooled donor culture, highlights that prolonged astilbin exposure is statistically associated with a coordinated, three-stage ecological succession within the simulated human gut microbiota. The system progressed from an initial transitional phase, through a putative D3 metabolic transition point, to a late-stage stabilized configuration. Importantly, this structural succession correlated with a marked metabolic shift away from markers associated with proteolytic fermentation toward the robust accumulation of 3-IPA and enhanced predicted antioxidant potential. Given the methodological limitations of a single pooled continuous culture without a vehicle control, these findings should be interpreted cautiously as an associative, hypothesis-generating temporal framework. This study sets the stage for future individual-specific in vivo models and targeted mechanistic experiments to definitively validate astilbin’s role as a dynamic ecological modulator.

## Data Availability

The data that support the findings of this study are available on request from the corresponding authors. The data are not publicly available owing to privacy or ethical restrictions.

## Supplementary Materials

The following supporting information can be downloaded at: https://www.mdpi.com/article/10.3390/nu18162616/s1, Figure S1: Key differential metabolites associated with astilbin intervention. Metabolites are conceptually categorized by their potential biological functions: (A–C) Tryptophan metabolic reprogramming, (D–F) Signature metabolism & nutrition, (G–I) Gut-liver axis & polyphenol biotransformation, and (J–L) Inflammation resolution & putrefaction suppression. Differential metabolites were identified based on the thresholds: Variable Importance in Projection (VIP) > 1, adjusted P (FDR) < 0.05, and |log_2_ Fold Change (FC)| > 1.0 (or FC > 2). Statistical significance for specific metabolites versus D0 was evaluated using one-way ANOVA with Dunnett’s post hoc test (* *p* < 0.05, ** *p* < 0.01, *** *p* < 0.001); Figure S2: Metabolic shifts and multivariate statistical validation associated with the D3 transition phase and D7 terminal state. (A,B) Principal component analysis (PCA) (A) and orthogonal partial least squares discriminant analysis (OPLS-DA) (B) score plots comparing the Day 3 (D3) and Day 7 (D7) metabolomes, demonstrating significant metabolic separation. (C) Permutation test validation plot for the OPLS-DA model discriminating D3 and D7. (D) Volcano plot illustrating the significantly differential metabolites between D7 and D3. (E) Horizontal bar plot detailing the log_2_(FC) of representative core metabolites comparing D7 to D3. (F) Permutation test validation plot for the OPLS-DA model discriminating D3 and D0. (G) Permutation test validation plot for the OPLS-DA model discriminating D7 and D0; Table S1: Instrument conditions for the analysis of metabolites; Table S2: Composition and final concentrations of the mixed internal standards used for pseudo-targeted metabolomics.
