# Supplementary material for: Temporal Multi-Omics Reveals Microbial and Metabolic Succession Following Astilbin Treatment in a Human Colonic Model
Source: Nutrients. 2026 Aug 10;18(16):2616. doi: 10.3390/nu18162616 (PMC13516478; doi:10.3390/nu18162616)
Supplement: Supplementary file 1 [file nutrients-18-02616-s001.zip › nutrients-4444821-supplementary.pdf]

# Astilbin drives a three-stage microbial succession to reprogram colonic metabolism toward 3-IPA-driven homeostasis

**Tingwei Wang [1, 2], Chang Liu [3], Jian ji [3], Shuang Zhang [4], Shengfang Wu [4], Bangen Xia [5], Ruowei Xia [5], Nian Qu [1, 2], Maiqiu Wang [1, 2], Lei Zhang [1, 2*], Yongli Ye [3*]**

[1] College of Biological & Chemical Engineering, Zhejiang University of Science and Technology, Hangzhou 310023, Zhejiang, China

[2] Zhejiang Key Laboratory of Biomedical Intelligent Computing Technology, Zhejiang, China

[3] School of Food Science and Technology, International Joint Laboratory on Food Safety, Synergetic Innovation Center of Food Safety and Quality Control, Jiangnan University, Wuxi, Jiangsu 214122, P.R. China,

[4] Jiangnan University Instrumental Analysis and Laboratory Animal Center, Wuxi, Jiangsu, China,

[5] Ningbo Xiabang New Pharmaceutical Technology Co., Ltd., Ningbo, Zhejiang, 315000, China

Corresponding authors: Lei Zhang and Yongli Ye

Lei Zhang: E-mail address: leizhang@zust.edu.cn

Yongli Ye: E-mail address: yyly0222@jiangnan.edu.cn

Figure S1

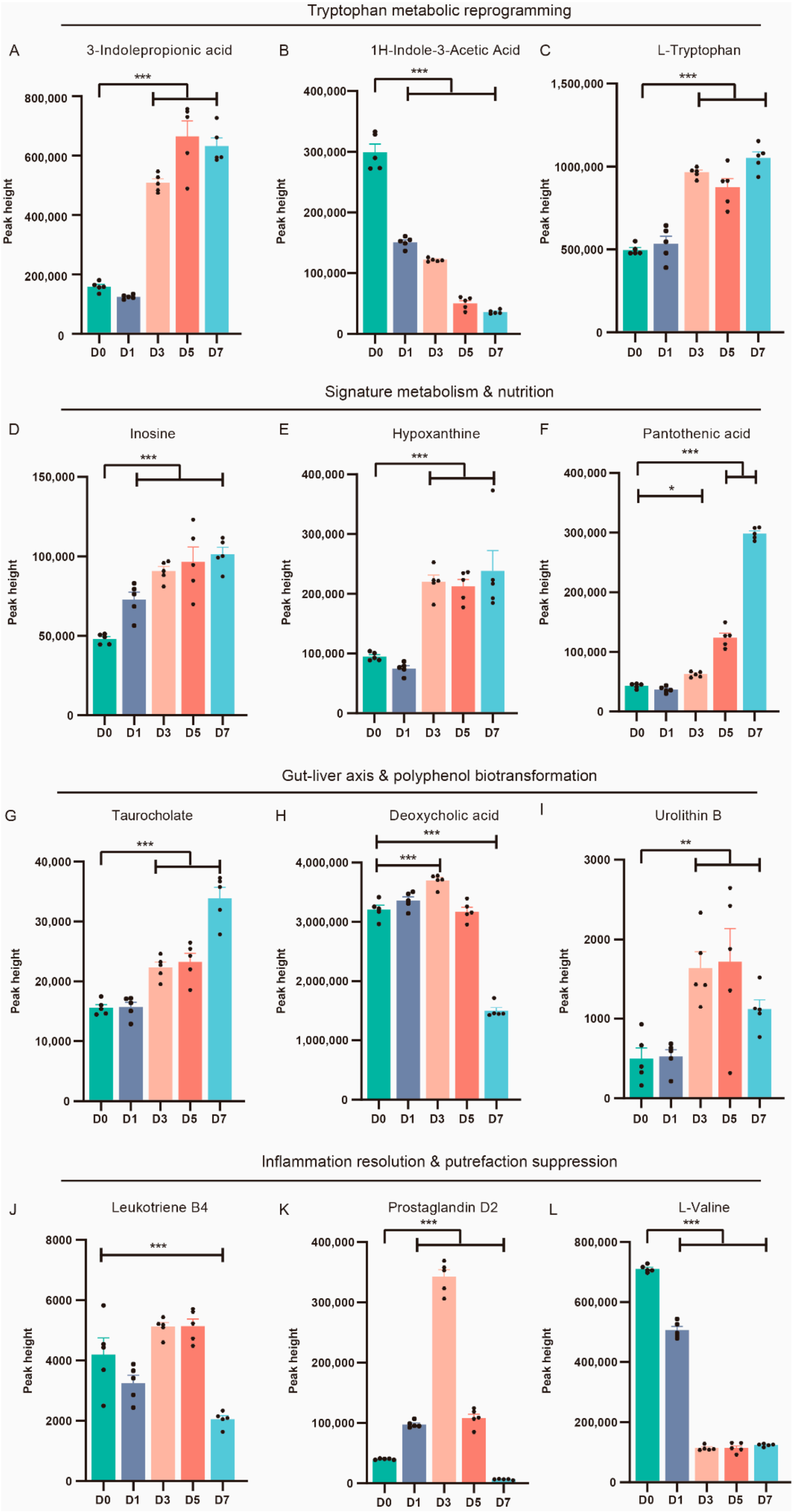

Tryptophan metabolic reprogramming
A
3-Indolepropionic acid
B
1H-Indole-3-Acetic Acid
C
L-Tryptophan
Signature metabolism & nutrition
D
Inosine
E
Hypoxanthine
F
Pantothenic acid
Gut-liver axis & polyphenol biotransformation
G
Taurocholate
H
Deoxycholic acid
I
Urolithin B
Inflammation resolution & putrefaction suppression
J
Leukotriene B4
K
Prostaglandin D2
L
L-Valine
Peak height
D0 D1 D3 D5 D7

# Figure S2

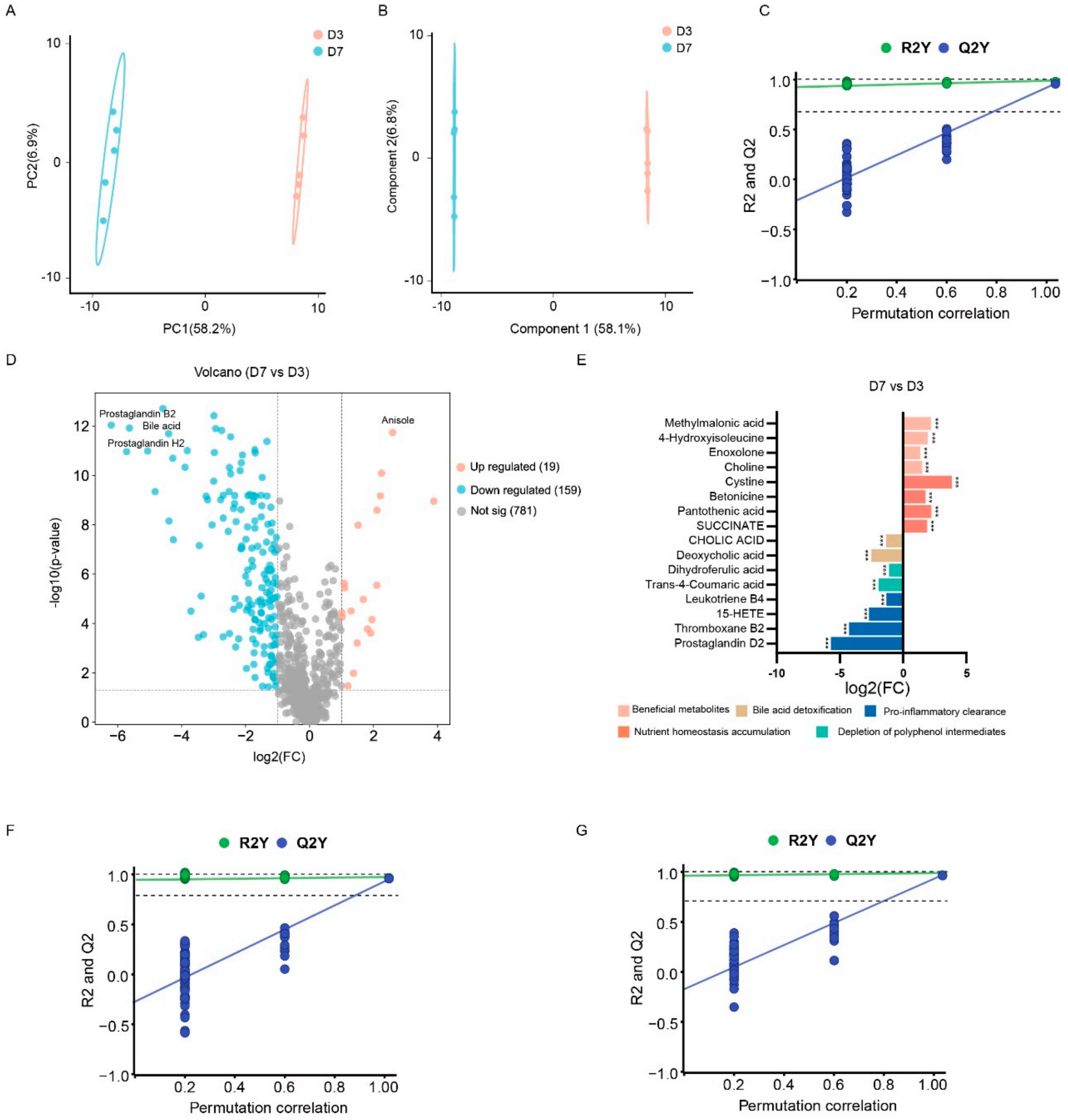
A
B
C
D
E
F
G
D3
D7
PC1(58.2%)
PC2(6.9%)
Component 1 (58.1%)
Component 2(6.8%)
R2Y
Q2Y
R2 and Q2
Permutation correlation
Volcano (D7 vs D3)
Prostaglandin B2
Bile acid
Prostaglandin H2
Anisole
Up regulated (19)
Down regulated (159)
Not sig (781)
-log10(p-value)
log2(FC)
D7 vs D3
Methylmalonic acid
4-Hydroxyisoleucine
Enoxolone
Choline
Cystine
Betonicine
Pantothenic acid
SUCCINATE
CHOLIC ACID
Deoxycholic acid
Dihydroferulic acid
Trans-4-Coumaric acid
Leukotriene B4
15-HETE
Thromboxane B2
Prostaglandin D2
log2(FC)
Beneficial metabolites
Bile acid detoxification
Pro-inflammatory clearance
Nutrient homeostasis accumulation
Depletion of polyphenol intermediates

# Figure Captions

**Figure S1** Key differential metabolites associated with astilbin intervention. Metabolites are conceptually categorized by their potential biological functions: **(A-C)** Tryptophan metabolic reprogramming, **(D-F)** Signature metabolism & nutrition, **(G-I)** Gut-liver axis & polyphenol biotransformation, and **(J-L)** Inflammation resolution & putrefaction suppression. Differential metabolites were identified based on the thresholds: Variable Importance in Projection (VIP) > 1, adjusted *P* (FDR) < 0.05, and $|\log_2$ Fold Change (FC)$|$ > 1.0 (or FC > 2). Statistical significance for specific metabolites versus D0 was evaluated using one-way ANOVA with Dunnett's post hoc test (* $P < 0.05$, ** $P < 0.01$, *** $P < 0.001$).

**Figure S2** Metabolic shifts and multivariate statistical validation associated with the D3 transition phase and D7 terminal state. **(A, B)** Principal component analysis (PCA) **(A)** and orthogonal partial least squares discriminant analysis (OPLS-DA) **(B)** score plots comparing the Day 3 (D3) and Day 7 (D7) metabolomes, demonstrating significant metabolic separation. **(C)** Permutation test validation plot for the OPLS-DA model discriminating D3 and D7. **(D)** Volcano plot illustrating the significantly differential metabolites between D7 and D3. **(E)** Horizontal bar plot detailing the $\log_2$(FC) of representative core metabolites comparing D7 to D3. **(F)** Permutation test validation plot for the OPLS-DA model discriminating D3 and D0. **(G)** Permutation test validation plot for the OPLS-DA model discriminating D7 and D0.

Table S1 Instrument conditions for the analysis of metabolites

<table>
<tr><th>Instrument conditions</th><th>AB Sciex QTRAP 5500 mass spectrometer</th></tr>
<tr><td>Spectrum Column</td><td>Waters Acquity BEH C8 column (100 mm × 2.1 mm, 1.7 μm)</td></tr>
<tr><td>Mobile Phase</td><td>Positive ion mode:<br>Mobile phase A: 0.1% formic acid in water<br>Mobile phase B: 0.1% formic acid in acetonitrile<br>Negative ion mode:<br>Mobile phase C: 6.5 mmol/L ammonium bicarbonate in water<br>Mobile phase D: 6.5 mmol/L ammonium bicarbonate in 95% methanol/water</td></tr>
<tr><td>Gradient Profile</td><td>positive ion mode
<table>
<tr><th>Time (min)</th><th>Percentage A (%)</th><th>Percentage B (%)</th><th>Flow rate (mL/min)</th></tr>
<tr><td>0</td><td>95</td><td>5</td><td>0.35</td></tr>
<tr><td>1</td><td>95</td><td>5</td><td>0.35</td></tr>
<tr><td>24</td><td>0</td><td>100</td><td>0.35</td></tr>
<tr><td>28</td><td>0</td><td>100</td><td>0.35</td></tr>
<tr><td>28.1</td><td>95</td><td>5</td><td>0.35</td></tr>
<tr><td>30</td><td>95</td><td>5</td><td>0.35</td></tr>
</table>
negative ion mode
<table>
<tr><th>Time (min)</th><th>Percentage A (%)</th><th>Percentage B (%)</th><th>Flow rate (mL/min)</th></tr>
<tr><td>0</td><td>98</td><td>2</td><td>0.35</td></tr>
<tr><td>1</td><td>98</td><td>2</td><td>0.35</td></tr>
<tr><td>18</td><td>0</td><td>100</td><td>0.35</td></tr>
<tr><td>22</td><td>0</td><td>100</td><td>0.35</td></tr>
<tr><td>22.1</td><td>98</td><td>2</td><td>0.35</td></tr>
<tr><td>25</td><td>98</td><td>2</td><td>0.35</td></tr>
</table>
</td></tr>
<tr><td>Injection Volume</td><td>5 μL</td></tr>
<tr><td>Mass Parameters</td><td>MRM mode<br>Mass range: 5 – 2000 m/z<br>Scan speed: ≥ 20,000 Da/sec<br>Polarity switching time: ≤ 5 ms<br>Detector dynamic range: > $10^6$<br>Curtain gas (CUR): 25 – 30 psi<br>Ion spray voltage (IS): +5500 V (positive) / –4500 V (negative)<br>Nebulizer gas (GS1): 40 – 80 psi<br>Source temperature (TEM): 400 – 500 °C<br>Declustering potential (DP): (+) 65 V / (–) 60 V<br>Entrance potential (EP): (+) 10 V / (–) 10 V</td></tr>
</table>

Table S2. Composition and final concentrations of the mixed internal standards used for pseudo-targeted metabolomics.

| Internal Standard | Final Concentration (ng/mL) |
| --- | --- |
| Carnitine C2:0-d3 | 60 |
| Carnitine C8:0-d3 | 37.5 |
| Carnitine C10:0-d3 | 37.5 |
| Carnitine C16:0-d3 | 56.5 |
| LPC 19:0 | 281.5 |
| FFA C16:0-d3 | 937.5 |
| FFA C18:0-d3 | 937.5 |
| CDCA-d4 | 562.5 |
| Trp-d5 | 1594 |
| Phe-d5 | 1350 |
| SM 12:0 | 281.5 |
| Choline-d4 | 750 |
| Cholic acid-d4 | 694 |
| Glutamic acid-d3 | 1500 |
